# Editorial: Role of imaging in biliary tract cancer: diagnosis, staging, response prediction and image-guided therapeutics

**DOI:** 10.3389/fonc.2024.1387531

**Published:** 2024-03-19

**Authors:** Pankaj Gupta, Avinash Kambadakone, Bhawna Sirohi

**Affiliations:** ^1^ Department of Radiodiagnosis and Imaging, Postgraduate Institute of Medical Education and Research, Chandigarh, India; ^2^ Department of Radiology, Massachusetts General Hospital, Harvard Medical School, Boston, MA, United States; ^3^ Department of Medical Oncology, BALCO Medical Centre, Raipur, Chhattisgarh, India

**Keywords:** staging, biliary tract cancer (BTC), gallbladder cancer (GBC), computed tomography, radiomics, endoscopy, ERCP (cholangiopancreatography, endoscopic retrograde)

Biliary tract cancers (BTC) comprise a spectrum of cancers arising from the intrahepatic or extrahepatic biliary tree (cholangiocarcinoma, CCA) and gallbladder (gallbladder cancer, GBC) (1). There is marked geographical variation in the incidence of BTCs (1). The incidence of CCA and GBC is low in high-income countries (2–4). The incidence of CCA is 40 times higher in endemic regions of China and Thailand (5). GBC incidence is the highest in women in Southern Chile and Northern India (6).

Recent studies have shown that CCA arises from two types of stem cells that determine its radiological appearance and prognosis (7, 8). CCA is classified anatomically into intrahepatic CCA (iCCA) and extrahepatic CCA (perihilar and distal CCA) (9). Perihilar CCA is classified most commonly based on the longitudinal extent of the disease (Bismuth Corlette system). However, classification systems that consider vascular involvement, remnant liver volume, lymph node, and distant metastases (TNM, MSKCC, Deoliveira) allow better resectability assessment and prognostication (10).

The early diagnosis of BTC is challenging due to non-specific symptoms (11). Most patients have advanced unresectable disease at the time of diagnosis. The imaging appearance of iCCA depends on the potential cell of origin (12). Large and small duct types of ICCs have different morphological appearances (**Figure 1**). Perihilar CC presents most commonly as a periductal infiltrating lesion, which must be differentiated from benign strictures (13). GBC manifests as a mass-replacing the gallbladder, wall-thickening, combined form, or intraluminal polypoidal form (14). Each form poses a distinct challenge in differentiation from benign diseases (15). Ultrasound (US), computed tomography (CT), and magnetic resonance imaging (MRI) are the most common imaging tests employed for the detection of BTC. US-based risk stratification systems, e.g., gallbladder reporting and data system (GB-RADS), guide the utilization of further imaging tests in patients with gallbladder lesions (16, 17). Multiphasic contrast-enhanced CT allows accurate staging of BTC (18). MRI and magnetic resonance cholangiopancreatography (MRCP) are preferred to evaluate the longitudinal extent of perihilar CC, determination of local extent and intrahepatic metastases. A multiparametric MRI has been recently proposed to characterize gallbladder wall thickening (19). [^18^F]-2-fluoro-2-deoxy-d-glucose–positron emission tomography (FDG PET) is not recommended for primary diagnosis of BTC (20). FDG PET may allow accurate diagnosis of nodal metastases, distant metastases, and recurrence (21). Fibroblast-activated protein (FAP) is highly expressed in cancer-associated fibroblasts. Gallium 68 (^68^G)-FAP inhibitor (FAPI) is a molecular target of FAP. FAPI PET enables the detection of small primary or metastatic lesions with a strong desmoplastic reaction. 68G-FAPI PET has been reported to be superior to FDG-PET in detecting various primary tumors (22). In this Research Topic, Ouyang et al. reported a systematic review comparing ^19^F-FDG PET with ^68^G-FAPI-04 PET in primary digestive system malignancy. They reviewed 15 studies comprising 383 patients and reported higher pooled sensitivity (98% vs. 73%) and specificity of 68Ga-FAPI-04 PET (0.81 vs. 0.77) compared to FDG PET for gastric, liver, biliary tract, and pancreatic cancers.

**Figure 1:**
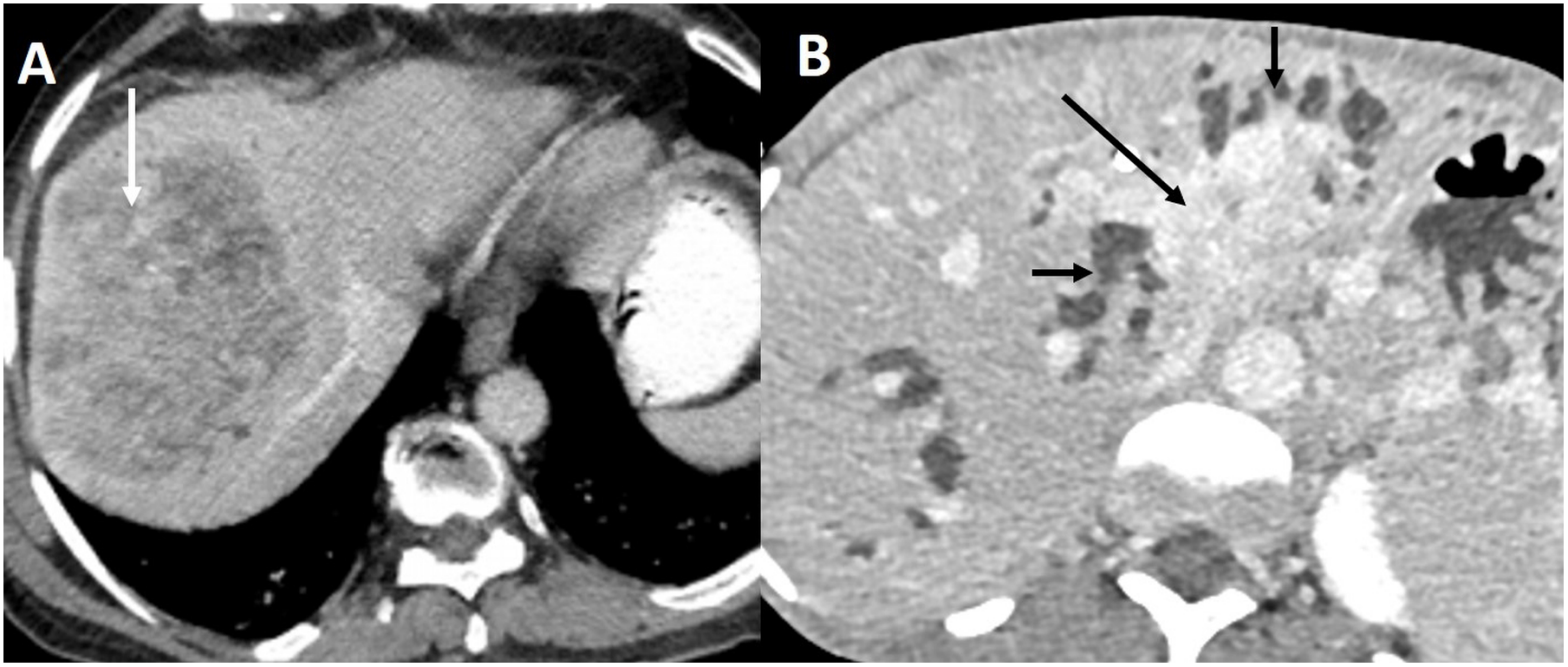
Imaging appearance of small and large duct intrahepatic cholangiocarcinoma. **(A)** Contrast-enhanced CT in the portal venous phase shows small duct ICC as a heterogeneously enhancing mass (arrow) in the periphery of the liver without bile duct dilatation. **(B)** Large duct ICC appears as an enhancing mass (arrow) infiltrating the left sided bile ducts causing biliary dilatation (short arrows) in the delayed phase CT.

Premalignant bile duct lesions include biliary intraepithelial neoplasm (BiN) and intraductal papillary neoplasm of the bile duct (IPNB) (23). Bile duct adenoma (BDA) is a rare benign bile duct lesion and is considered a controversial precursor to ICC (24). Pre-operative diagnosis of BDA is challenging. Huang et al. reported the imaging appearance of 32 pathologically proven BDAs. The most common site was the common bile duct (68.7%), and the most common morphology was focal eccentric mass (43.7%), followed by plaque-like masses (28.1%). BDAs with CC were associated with infiltrative masses. Dynamic contrast-enhanced MRI (18 lesions) showed moderate persistent enhancement in most lesions. The accurate diagnosis of BTC and differentiation from other bile duct lesions may necessitate advanced imaging techniques (25). Deng et al. reported a case where they used a single-operator peroral cholangioscopy system (SpyGlass) for diagnosing extrahepatic bile duct stricture seen at CT and MRI. Endoscopic retrograde cholangiopancreatography (ERCP) and cholangiogram revealed an indeterminate stricture. SpyGlass revealed an oval lesion with smooth, overlying mucosa. Extrahepatic biliary cystadenoma was confirmed at surgery. Zhu et al. compared the diagnostic accuracy of cytobrush, ERCP-guided biopsy, SpyGlass direct visual impression, and SpyGlass-guided biopsy (SpyBite) in the differentiation of benign and malignant biliary strictures in 1008 patients. The highest sensitivity was reported for SpyGlass (100%), followed by SpyBite (61.5%). However, the specificity of SpyGlass (55.6%) was significantly inferior to other methods (99-100%).

Identifying novel molecular targets may improve survival in patients with locally advanced BTC (26). Actionable molecular aberrations have been reported in up to 40% of ICC (27). The most promising molecular targets in ICC are isocitrate dehydrogenase 1 (IDH1) and fibroblast growth factor receptor 2 (FGFR2) (28). Several studies have reported a favorable prognosis in patients with IDH1 and FGFR 2 alterations (29–32). Brandi et al. reviewed the literature on the role of IDH and FGFR molecular alterations as positive prognostic markers in CC. They highlighted the strengths and pitfalls of the available literature and concluded that better-designed trials are needed to provide conclusive evidence on the prognostic role of IDH and FGFR. Imaging studies may give a clue to the type of molecular aberration in ICC. Small bile duct ICCs are characterized by IDH and FGFR2 fusions (33). On the other hand, large duct ICC and extrahepatic CC show a high frequency of KRAS and TP53 gene mutations (33). Although there is relatively limited data on GBC, a precision medicine strategy is supported by some recent studies (34). Comprehensive genomic profiling of 760 GBC patients identified at least one actionable genetic aberration in 86.6% (35). The most frequent actionable gene alteration was CKDN2A, followed by ERBB2/HER-2. Anti-HER2-directed therapy may improve outcomes in unresectable GBC (35).

Imaging studies provide data for downstream tasks relevant to diagnosis, prognostication, or identification of gene mutations (36). Radiomics is one approach that extracts many quantitative features from radiological images (37). Radiomic data can also be used for tasks based on artificial intelligence (AI) (38). There are several studies on using radiomics and AI in BTC (39–46). Chen et al. extensively reviewed the current status of radiomics in ICC. They highlighted studies utilizing radiomics to predict lymph node metastases, microvascular invasion, early recurrence after surgery, prediction of survival, and differentiation of ICC from other liver tumors.

In conclusion, imaging plays a vital role in the detection and staging of BTC and also serves as a novel biomarker for response prediction. Imaging-based radiomics and AI can potentially impact outcomes in patients with BTC.

## Author contributions

PG: Conceptualization, Writing – original draft, Writing – review & editing. AK: Conceptualization, Writing – review & editing. BS: Conceptualization, Writing – review & editing.
